# High-Resolution Imaging Insights into Shoulder Joint Pain: A Comprehensive Review of Ultrasound and Magnetic Resonance Imaging (MRI)

**DOI:** 10.7759/cureus.48974

**Published:** 2023-11-17

**Authors:** Bhagyasri Nunna, Pratap Parihar, Mayur Wanjari, Neha Shetty, Nikita Bora

**Affiliations:** 1 Radiology, Jawaharlal Nehru Medical College, Datta Meghe Institute of Higher Education & Research, Wardha, IND; 2 Research and Development, Jawaharlal Nehru Medical College, Datta Meghe Institute of Higher Education & Research, Wardha, IND

**Keywords:** treatment, diagnosis, magnetic resonance imaging (mri), ultrasound, high-resolution imaging, shoulder joint pain

## Abstract

Shoulder joint pain is a complex and prevalent clinical concern affecting individuals across various ages and lifestyles. This review delves into the pivotal role of high-resolution imaging techniques, namely ultrasound and magnetic resonance imaging (MRI), in the comprehensive assessment and management of shoulder joint pain. We explore the anatomical foundations of the shoulder, common etiologies of pain, and the significance of precise diagnosis. High-resolution imaging facilitates the identification of various shoulder pathologies and is crucial in treatment planning, surgical interventions, and long-term prognosis assessment. We examine emerging technologies, discuss challenges and limitations, and chart potential future developments, emphasizing the ongoing evolution of imaging in this critical healthcare domain. In conclusion, high-resolution imaging is an indispensable tool, continually advancing to meet the diagnostic and therapeutic needs of individuals grappling with shoulder joint pain.

## Introduction and background

The shoulder joint is a highly complex and mobile joint that plays a pivotal role in our daily activities, allowing us to perform a wide range of motions, from simple tasks like reaching for an object to more complex activities such as throwing a baseball. However, this remarkable flexibility also makes the shoulder uncomfortable and painful. Shoulder joint pain is a common complaint among individuals of all ages and backgrounds and can significantly impact one's quality of life. This comprehensive review delves into the intricacies of shoulder joint pain, focusing on the pivotal role that high-resolution imaging, particularly ultrasound and magnetic resonance imaging (MRI), plays in its diagnosis and management [1,2]. The shoulder joint, or glenohumeral joint, is a ball-and-socket joint composed of the scapula's humeral head and the glenoid cavity. This joint is surrounded by a complex network of tendons, ligaments, and muscles, collectively known as the rotator cuff, which provides stability and allows for an extensive range of motion. However, the intricate nature of the shoulder joint also makes it susceptible to various injuries and conditions that can lead to pain [3].

Among the primary contributors to shoulder pain are rotator cuff injuries, encompassing tears or strains in the tendons that can arise from a spectrum of causative factors, including overuse, traumatic incidents, or age-related degenerative changes. Labral tears, another frequently observed source of discomfort, entail damage to the fibrocartilaginous rim (labrum) encircling the glenoid socket, often associated with individuals engaging in repetitive overhead activities. Metabolic disorders, including hyperuricemia, type 2 diabetes mellitus (T2DM), and thyroid diseases, as well as post-myocardial infarction (MI) and cerebrovascular accident (CVA), can contribute to shoulder-related complications. Arthritis is a prevalent concern affecting the shoulder joint, with conditions like osteoarthritis, rheumatoid arthritis, and other arthritic ailments causing chronic pain and limiting mobility. Additionally, inflammation-induced tendinitis and bursitis, often stemming from overexertion or trauma, are common causes. Traumatic incidents can also lead to fractures of the humerus or collarbone and dislocations of the shoulder joint, resulting in acute pain and instability. Shoulder pain may also arise from nerve compression disorders such as thoracic outlet syndrome and nerve impingement, potentially causing radiating pain. Furthermore, systemic conditions originating from the neck or spine and infrequent occurrences such as tumors can all present as shoulder pain. This highlights the multifaceted nature of this clinical complaint [4,5].

Accurate diagnosis is the bedrock for effectively managing shoulder joint pain, a complex and multifaceted clinical concern. While traditional clinical examinations and X-rays offer initial insights, their limitations in fully revealing the intricacies of shoulder pathologies necessitate the adoption of high-resolution imaging techniques, notably ultrasound and MRI [6]. High-resolution imaging confers numerous pivotal advantages in the evaluation of shoulder joint pain. First, it provides detailed and multidimensional visualizations of the shoulder joint and its adjacent structures, empowering clinicians to identify even the most subtle abnormalities. Second, these imaging modalities enable the precise differentiation of soft tissues, bones, and fluids, significantly enhancing the accuracy of diagnoses across a broad spectrum of conditions. Moreover, the non-invasive nature of high-resolution imaging diminishes the need for exploratory surgical procedures, thereby reducing associated risks and patient discomfort. Furthermore, the findings from high-resolution imaging inform critical treatment decisions, whether they involve conservative approaches like physical therapy or surgical interventions such as arthroscopy or joint replacement. Lastly, the serial use of imaging facilitates the monitoring of condition progression, aids in assessing treatment efficacy, and allows for timely adjustments, ensuring patients receive tailored and optimal care throughout their recovery journey. In conclusion, high-resolution imaging, represented by ultrasound and MRI, is transformative in enhancing the precision and efficacy of diagnosis and treatment strategies for shoulder joint pain, ultimately improving patient outcomes and overall quality of life [7,8].

This comprehensive review explores the role of high-resolution imaging, specifically ultrasound and MRI, in evaluating and managing shoulder joint pain. We will delve into each imaging modality's unique advantages and limitations, their application in diagnosing common shoulder pathologies, and their implications for treatment planning and patient outcomes. Throughout this review, we will also discuss emerging technologies and advancements in high-resolution imaging, potential future developments, and the challenges and limitations associated with these techniques. By the end of this review, readers should gain a comprehensive understanding of the pivotal role that high-resolution imaging plays in unraveling the complexities of shoulder joint pain and facilitating effective clinical decision-making.

## Review

Anatomy of the shoulder joint

Brief Overview of Shoulder Anatomy

The shoulder, a complex and highly mobile joint, relies on a sophisticated interplay of bones, muscles, and connective tissues to perform its myriad functions. At the heart of this intricate system is the humerus, the upper arm bone, which forms the central axis of the shoulder joint. Its rounded head articulates with the glenoid cavity of the scapula, creating a pivotal ball-and-socket joint that permits a wide range of arm movements [3]. The scapula, commonly known as the shoulder blade, serves as the foundational framework for the shoulder joint. This flat, triangular bone boasts essential landmarks, including the glenoid fossa or cavity, a shallow, concave depression where the humeral head securely attaches. Additionally, the acromion and coracoid processes, acting as anchoring points for muscles and ligaments, play a vital role in the shoulder's stability and function [3].

Situated on the scapula, the glenoid cavity is a critical component of the shoulder joint, providing the primary point of articulation for the humeral head. This structural feature is pivotal in maintaining the joint's stability and enabling its impressive mobility [3]. The integrity and function of the shoulder joint are heavily reliant on the rotator cuff, a group of four muscles, and their associated tendons. These muscles, known as the supraspinatus, infraspinatus, teres minor, and subscapularis, envelop the joint, contributing to its stability and a wide array of movements.

The labrum, a fibrocartilaginous rim that encircles the glenoid cavity, enhances the joint's stability and provides essential cushioning. This anatomical feature is crucial for proper shoulder function as it helps anchor the humeral head and provides added support [3]. Small fluid-filled sacs, known as bursae, are strategically positioned between tendons and bones to facilitate the intricate movements of the shoulder joint. These bursae act as lubrication and cushioning devices, reducing friction and allowing for smooth motion [3]. Finally, various ligaments, such as the coracohumeral and glenohumeral ligaments, contribute significantly to the stability of the shoulder joint. These connective tissues play a vital role in connecting bones and reinforcing the joint's structural integrity, ensuring that it can perform its complex tasks with precision and stability [3].

Structures Involved in Shoulder Joint Pain

Shoulder joint pain can arise from many structures within the complex shoulder anatomy, each playing a distinct role in its function and vulnerability to injury. One of the primary culprits is the rotator cuff, a group of tendons and muscles critical for shoulder stability and mobility. Rotator cuff injuries, including tears, tendinitis, and impingement, can commonly affect athletes, laborers, and those experiencing age-related degeneration. The integrity of these tendons is paramount for maintaining pain-free shoulder movement [9].

The labrum, a ring of cartilage that deepens the shoulder socket, also plays a pivotal role. Labral tears or damage can result from various factors, such as traumatic injuries, chronic overuse, or joint instability, causing pain and restricting the shoulder's range of motion. A healthy labrum enhances shoulder stability [10]. The articular surfaces of the shoulder joint are susceptible to arthritis, leading to pain, inflammation, and reduced joint function. Osteoarthritis, typically associated with age-related wear and tear, and rheumatoid arthritis, an autoimmune disorder affecting multiple joints, are common culprits [11].

Bursae, small fluid-filled sacs strategically positioned within the shoulder, can become inflamed, causing a condition known as bursitis. Inflammation of these sacs often results in localized pain and discomfort, with subacromial bursitis being a frequent companion to rotator cuff disorders, further contributing to shoulder pain [12]. Traumatic incidents like humerus fractures or shoulder joint dislocations can result in acute and severe pain, necessitating immediate medical attention due to the potential for complications and long-term consequences [13].

Nerve compression disorders, such as thoracic outlet syndrome or nerve impingement, can radiate shoulder, neck, and arm pain. These conditions often arise from the compression or irritation of nerve structures within the shoulder region, adding to the complexity of shoulder pain [14]. Beyond the rotator cuff, other muscles and tendons in the shoulder region can also contribute to discomfort when injured or overworked. Muscles like the deltoid, biceps, and triceps can become sources of pain and reduced function [15]. It is important to note that shoulder joint pain can occasionally originate from systemic conditions, referred pain from the neck or spine, or even tumors. These cases underscore the necessity of a comprehensive evaluation when diagnosing shoulder pain as its origins can be multifaceted and, at times, less apparent [5].

Diagnostic imaging modalities

Ultrasound

Principles and advantages: Ultrasound offers a range of compelling advantages in the field of medical imaging. One of its most remarkable features is its real-time imaging capacity, allowing clinicians to witness the shoulder joint in dynamic motion. This capability provides invaluable insights into the intricate movements of tendons, potential impingements, and the identification of dynamic instability issues. Moreover, ultrasound enables visualization of structural changes during activities that may trigger or worsen pain, ultimately facilitating precise and timely diagnoses [16]. Another key attribute of ultrasound is its non-invasive nature. Unlike some imaging modalities, it does not expose patients to ionizing radiation, ensuring their safety during repeated examinations. This inherent safety factor makes ultrasound a preferred choice, particularly when continuous monitoring is essential for effective healthcare management [17].

Additionally, the widespread availability of ultrasound equipment in various healthcare settings contributes significantly to its appeal. This accessibility makes it a cost-effective option and ensures that patients can benefit from its diagnostic capabilities without encountering significant delays or logistical challenges. Ultrasound's ubiquity in medical facilities enhances its utility and ease of use [18]. Furthermore, the modern ultrasound machines of today boast impressive technological advancements, offering high-resolution imaging capabilities. This high level of detail enables healthcare professionals to examine the shoulder joint precisely, revealing intricate structures such as soft tissues, tendons, ligaments, and bursae. Such detailed visualization is indispensable for identifying various pathologies and understanding contributing factors to patients' shoulder issues [19].

Limitations: While ultrasound offers numerous advantages in medical imaging, it has limitations. Operator proficiency plays a crucial role in the quality of ultrasound images. The expertise and experience of the operator significantly impact the accuracy of the diagnostic information obtained. Achieving optimal image quality and accurately interpreting ultrasound findings necessitate a high degree of skill in musculoskeletal imaging. Therefore, the variability in operator skill levels can introduce inconsistencies in the reliability of the results [20].

Another limitation of ultrasound is its challenge in visualizing deep-seated structures, especially in cases where there is a considerable amount of soft tissue between the ultrasound probe and the target area. This limitation can be particularly pronounced in individuals with larger body sizes or when attempting to examine deep structures. In such situations, achieving adequate penetration to visualize these deeper anatomical regions may be more challenging, potentially compromising the comprehensiveness of the examination [21].

Patient cooperation is another essential factor that can affect the effectiveness of ultrasound imaging. In severe pain, limited range of motion, or discomfort, patients may find it difficult to tolerate the necessary positioning and movement during the examination. This can hinder the operator's ability to obtain clear and comprehensive images as patient discomfort may limit their ability to cooperate fully during the procedure. Therefore, the success of ultrasound examinations relies on the patient's ability to participate comfortably and depends on the experience of the sonologist in shoulder-hand pathology [22].

Magnetic Resonance Imaging (MRI)

Principles and advantages: MRI is a powerful diagnostic tool, particularly when assessing the intricacies of the shoulder joint and related structures. One of its standout features is its exceptional soft tissue contrast. MRI's ability to provide a high level of contrast between various soft tissues makes it exceptionally well-suited for scrutinizing the complex anatomy of the shoulder. This capability facilitates the precise visualization and differentiation of structures such as muscles, tendons, ligaments, and bursae, which is pivotal in identifying pathologies and contributing factors [7]. Moreover, MRI offers the advantage of multiplanar imaging. This means it can capture images of the shoulder joint from multiple angles and orientations. This comprehensive, multiplanar approach significantly enhances diagnostic accuracy by allowing healthcare providers to assess structures from various perspectives. As a result, subtle abnormalities that might not be apparent in a single plane can be detected, contributing to a more thorough evaluation [7].

Another compelling benefit of MRI is its non-ionizing nature. Unlike some imaging modalities, MRI does not involve ionizing radiation, ensuring the safety of patients. This characteristic makes MRI a highly desirable option, particularly for individuals who may require repeated imaging examinations or have concerns about radiation exposure. The absence of ionizing radiation is a significant advantage regarding patient safety and long-term health considerations [23]. Furthermore, MRI's versatility extends beyond the shoulder joint alone. It can encompass whole-body imaging, allowing healthcare providers to evaluate structures beyond the immediate shoulder region. This broader imaging scope can be invaluable when searching for underlying causes of shoulder pain that may originate from adjacent areas or have systemic implications. This capability enables a more comprehensive assessment of a patient's condition, aiding in identifying complex, interconnected issues [24].

Limitations: MRI is a valuable tool in medical diagnostics, but it has its considerations and limitations. One significant aspect is the cost and accessibility of MRI technology. MRI machines represent substantial financial investments, and their availability is less widespread than ultrasound machines. This limited accessibility can potentially create barriers to healthcare access for certain patients, especially in regions with limited resources, where the cost of MRI may be a considerable burden for both healthcare systems and individuals [25].

Patient cooperation is another crucial factor to consider when opting for MRI examinations. Unlike some imaging modalities, a successful MRI procedure requires patients to remain perfectly still for an extended period, ranging from 15 minutes to over an hour. This demand for immobility can be particularly challenging for individuals with severe pain, claustrophobia, or conditions limiting their mobility. In such cases, patient comfort and their ability to comply with the examination requirements can significantly impact the quality and success of the imaging results [26].

Furthermore, contrast agents, such as gadolinium, are sometimes necessary to enhance the visibility of specific structures or abnormalities during MRI scans. However, it is important to note that not all patients are suitable candidates for contrast agents. Individuals with underlying kidney problems or allergies to the contrast material may face potential risks, necessitating a careful evaluation of each patient's medical history before deciding to use these agents. Balancing the benefits of enhanced imaging with the patient's safety is a critical consideration in the MRI process [27].

Comparative Analysis of Ultrasound and MRI

Choosing between ultrasound and MRI for evaluating shoulder joint pain often depends on the clinical scenario, patient characteristics, and the required information. Ultrasound excels in real-time assessment and is particularly useful for dynamic studies. It is readily available and cost-effective. In contrast, MRI offers superior soft tissue contrast and is ideal for a comprehensive assessment of the shoulder joint's anatomy, making it a valuable tool for complex cases or preoperative planning [28]. The choice between the two modalities may also depend on factors such as the patient's condition, the suspected pathology, and the clinician's expertise. In many cases, a combination of ultrasound and MRI may be employed to provide a holistic assessment of shoulder joint pain, leveraging the strengths of each modality to arrive at an accurate diagnosis and treatment plan [29].

Common causes of shoulder joint pain

Rotator Cuff Tears

Overuse: Prolonged engagement in repetitive overhead activities, such as throwing, lifting heavy objects, or participating in sports that involve constant shoulder motion, can contribute to the gradual wear and tear of the rotator cuff tendons. Over time, this wear and tear may culminate in the development of tears within the tendon fibers [30].

Trauma: Acute injuries, such as a fall onto an outstretched arm, a direct blow to the shoulder, or a sudden forceful motion, can result in immediate and traumatic tears within the rotator cuff tendons. These injuries are often associated with a distinct onset of pain and functional impairment [31].

Age-related degeneration: The tendons of the rotator cuff are not immune to the effects of aging. As individuals age, these tendons may undergo degenerative changes, including tissue weakening and alterations in collagen composition. These age-related degenerative changes can render the tendons more vulnerable to tears, even without explicit trauma or overuse [32].

Position: The positioning of the upper limb during sleep is also a significant factor contributing to mild to moderate pain. Sleeping with the upper arm elevated above the head or consistently on one side, particularly in a lateral position for an extended period, can lead to discomfort. Additionally, intra-articular dehydration may exacerbate this discomfort [32].

Labral Tears

Trauma: Labral tears can occur due to direct trauma to the shoulder. This may involve a forceful blow to the shoulder joint or a shoulder dislocation event, which can lead to labral damage. Traumatic labral tears are often present with a distinct onset of pain and functional limitations [33].

Overuse: Athletes and individuals who engage in repetitive overhead activities are at a heightened risk of developing labral tears over time. Sports such as baseball pitching, swimming, and volleyball involve frequent and intense shoulder motions, contributing to the labrum's wear and tear. Overuse-induced labral tears may manifest gradually, increasing pain and discomfort [34].

Instability: Shoulder instability, characterized by the partial or complete dislocation of the humeral head from the glenoid cavity, can lead to labral injuries. In cases of instability, the labrum may become stretched, torn, or detached as the shoulder joint experiences abnormal movements. Individuals with recurrent shoulder instability are particularly prone to labral tears [10].

Arthritis

Osteoarthritis: Osteoarthritis, often called "wear and tear" arthritis, develops gradually over time. It occurs due to the progressive breakdown of the shoulder joint's protective cartilage. As cartilage deteriorates, the joint experiences increased friction and bone-on-bone contact, leading to pain, inflammation, and reduced range of motion. Osteoarthritis is a chronic, aching shoulder pain that worsens with activity and may accompany joint stiffness [35].

Rheumatoid arthritis: Rheumatoid arthritis is an autoimmune disorder in which the body's immune system mistakenly attacks the synovium, the lining of the joint capsule. This inflammation can result in pain, swelling, and joint damage in the shoulder joint. Rheumatoid arthritis often affects multiple joints throughout the body and can cause systemic symptoms and localized joint pain. Shoulder pain related to rheumatoid arthritis is typically characterized by aching, stiffness, and joint swelling [36].

Post-traumatic arthritis: Post-traumatic arthritis develops following a traumatic injury to the shoulder, such as a fracture or dislocation. These injuries can disrupt the typical joint structure and lead to cartilage damage. Over time, the compromised joint may develop arthritis, resulting in chronic shoulder pain and functional impairment. Post-traumatic arthritis can manifest symptoms like osteoarthritis, pain, stiffness, and limited mobility [37].

Tendinitis and Bursitis

Overuse: Overusing the shoulder joint primarily contributes to tendinitis and bursitis. Repetitive movements or activities that subject the tendons and bursae to excessive stress and strain, such as lifting heavy objects, performing overhead work, or engaging in sports with repetitive shoulder motion (e.g., swimming or baseball pitching), can provoke inflammation and irritation. This overuse-related inflammation is often called "wear and tear" [38].

Infection: In some instances, tendinitis and bursitis can be initiated by bacterial infection, leading to a condition known as septic bursitis. While septic bursitis is less common than non-infectious forms, it is a severe medical concern that requires prompt intervention. Bacterial infection can infiltrate the bursae, causing acute inflammation and often leading to systemic symptoms such as fever and chills [39].

Fractures and Dislocations

Shoulder injuries stemming from various mechanisms are common, often resulting in fractures or dislocations requiring medical attention. One prevalent cause of such injuries is falls, where individuals land on an outstretched arm. This mechanism of injury can lead to significant trauma to the shoulder joint, resulting in damage to the bones and the surrounding structures [40]. Sports-related activities, particularly high-impact sports like football, skiing, or contact sports, present another substantial risk for shoulder trauma. Collisions with other players, falls, or awkward landings can subject the shoulder to considerable force, potentially resulting in fractures or dislocations that require medical intervention [41]. With their potential for generating substantial force, motor vehicle accidents pose a significant risk to the shoulder region. Sudden deceleration or direct impact during a collision can lead to fractures of the humerus, clavicle, or shoulder joint dislocations. These injuries are not uncommon in the aftermath of motor vehicle accidents and necessitate prompt medical evaluation and care [42].

Other Causes

Nerve compression: Conditions such as thoracic outlet syndrome or nerve impingement can lead to radiating pain that originates in the neck, upper back, or chest and extends into the shoulder and arm. Nerve compression syndromes can result from anatomical variations or structural abnormalities that compress nerves or blood vessels in the shoulder region. These conditions may cause numbness, tingling, weakness, and pain [43].

Referred pain: Pain originating from other body regions, such as the neck, cervical, or thoracic spine, can manifest as shoulder pain. This phenomenon, referred to as pain, can pose diagnostic challenges as the source of pain may not be immediately evident. Healthcare providers must evaluate comprehensively to differentiate between shoulder-related pain and referred pain from other anatomical structures [44].

Tumors: Although relatively rare, tumors in the shoulder region can lead to localized pain and discomfort. Tumors may originate from the bone (bone tumors), soft tissues (soft tissue sarcomas), or other structures within the shoulder. It is essential to evaluate any suspicious or unexplained shoulder pain through imaging and, in some cases, biopsy to rule out the presence of tumors [45].

Systemic conditions: Certain systemic conditions and diseases can result in widespread musculoskeletal pain, including in the shoulder region. Conditions such as diabetes and fibromyalgia are examples of disorders that can cause diffuse, chronic pain. Managing shoulder pain in systemic conditions often involves a multidisciplinary approach, addressing the underlying condition and localized symptoms [46].

Role of high-resolution imaging in diagnosis

The role of high-resolution imaging in diagnosing shoulder joint conditions is essential for several reasons. It allows healthcare professionals to accurately identify the underlying causes of pain and dysfunction in the shoulder, which is crucial for developing an effective treatment plan. Two commonly used high-resolution imaging techniques for diagnosing shoulder joint conditions are ultrasound and MRI [47].

Ultrasound Imaging

Dynamic imaging: Ultrasound stands out in its ability to provide dynamic images, granting healthcare providers the capacity to assess the shoulder joint in motion. This dynamic capability is invaluable when evaluating tendon and muscle functionality and pinpointing conditions like rotator cuff tears and impingement syndromes [48].

Real-time assessment: Another noteworthy advantage of ultrasound is its capacity for real-time assessment. It allows immediate visualization of structures, making it a valuable tool for guiding injections and other interventional procedures in real-time. This real-time feedback not only aids in accurate diagnosis but also enhances the precision of treatment interventions, contributing to improved patient outcomes [49].

Evaluation of soft tissues: Ultrasound evaluates soft tissues, including tendons, ligaments, and muscles. It can identify abnormalities within these structures, such as tears, inflammation, or calcifications. This detailed soft tissue assessment is crucial for diagnosing and managing a wide range of shoulder-related conditions, ensuring comprehensive patient care [50].

Magnetic Resonance Imaging

MRI offers several advantages in evaluating the shoulder joint, making it a valuable tool in musculoskeletal imaging. One of its primary strengths is its ability to provide highly detailed anatomical information. With MRI, clinicians can obtain comprehensive shoulder images encompassing bones, cartilage, ligaments, tendons, and soft tissues. This level of detail allows for a thorough assessment of the entire shoulder complex, facilitating precise diagnosis and treatment planning [7].

Another key feature of MRI is its capacity for multiplanar imaging. By capturing images in multiple planes, MRI creates a three-dimensional view of the shoulder, offering a comprehensive perspective of the anatomical structures. This capability aids in detecting subtle abnormalities and provides a deeper understanding of the spatial relationships between different shoulder components, contributing to more accurate diagnoses [7].

Furthermore, MRI can utilize contrast agents to enhance visualization in certain cases. This is particularly advantageous for detecting conditions like labral tears or tumors, where using contrast agents can improve the clarity of the images, making it easier to identify specific lesions and abnormalities [51].

Moreover, MRI stands out in its ability to provide quantitative data. It can offer precise measurements regarding the size and extent of lesions and the degree of tissue damage. This quantitative information is invaluable for treatment planning and monitoring the progression of shoulder conditions over time, allowing for more targeted and effective patient care [52].

Specific findings in ultrasound

Tendon Abnormalities

Rotator cuff tears: Ultrasound is exceptionally well-suited for evaluating rotator cuff tears, common causes of shoulder pain and dysfunction. It provides real-time visualization of the rotator cuff tendons, allowing healthcare providers to assess the integrity of these tendons. When a tear is present, ultrasound can depict this by revealing disruptions in the tendon fibers. The extent of the tear, whether partial or full-thickness, can also be assessed, helping to guide treatment decisions. For example, a full-thickness tear may require surgical repair, while a partial tear might be managed conservatively [53].

Tendinitis: Tendinitis, or inflammation of the tendons, can be readily observed using ultrasound. Inflammation often leads to changes in the appearance of the affected tendon on ultrasound images. These changes include thickening of the tendon and increased echogenicity, which refers to the brightness or whiteness of the tendon on the ultrasound screen. These visual cues indicate the presence of inflammation and help diagnose conditions like rotator cuff tendinitis or bicipital tendinitis. Accurate identification of tendinitis is crucial for appropriate management, which may involve rest, physical therapy, or anti-inflammatory treatments [54].

Bursal Involvement

Bursitis: Bursitis is another common shoulder condition characterized by the inflammation of bursae, which are tiny sacs filled with fluid that reduce friction between tendons, ligaments, and bones. Ultrasound can detect signs of bursitis by visualizing the bursae and the structures around them. When bursitis is present, ultrasound may show fluid accumulation within the affected bursa, which appears as an anechoic (dark) area within the bursa. As observed through Doppler ultrasound, increased vascularity indicates inflammation and can be another valuable diagnostic marker for bursitis. Accurate diagnosis of bursitis helps healthcare providers determine appropriate treatment, including rest, anti-inflammatory medications, or therapeutic interventions such as ultrasound-guided injections [55].

Impingement

Impingement is when structures within the shoulder joint, such as tendons or bursae, become compressed or irritated, often due to abnormal contact with surrounding bone or other tissues. Subacromial impingement involves explicitly the compression of structures, particularly the rotator cuff tendons, as they pass through the subacromial space, which is a narrow area between the acromion (a bony projection of the shoulder blade) and the head of the humerus (upper arm bone) [56].

Labral Tears and Instability

Identification of superior labrum anterior to posterior (SLAP) tears: SLAP tears, which affect the superior (top) part of the labrum where the biceps tendon attaches, can be effectively detected using ultrasound. This imaging modality can pinpoint disruptions in this area, including detachment or fraying of the labrum. By strategically positioning the ultrasound probe, healthcare providers can visualize and assess the integrity of the labrum, facilitating the identification of SLAP tears with precision [57].

Dynamic evaluation: Ultrasound's real-time imaging capabilities prove especially advantageous when evaluating labral tears and assessing shoulder joint stability. Patients can actively move their shoulders during the examination, allowing healthcare providers to observe how the labrum and surrounding structures respond to different motions. This dynamic evaluation is particularly valuable as it can reveal abnormal mobility or instability in the joint, and specific shoulder positions or movements may provoke symptoms, aiding in the diagnostic process [58].

Biceps tendon assessment: Given the close association between SLAP tears and the biceps tendon's attachment to the labrum, ultrasound can serve as a valuable tool to assess the condition of the biceps tendon itself. It can provide insights into signs of tendonitis or degeneration, comprehensively evaluating the structures involved in SLAP tears [59].

Guidance for intervention: In certain cases, ultrasound can be pivotal in guiding diagnostic and therapeutic procedures. For instance, it can facilitate ultrasound-guided injections of contrast dye or medication, confirming a labral tear's presence and precise location. This information is invaluable for planning potential surgical repairs or other therapeutic interventions, ensuring targeted and effective treatment strategies [60].

Complement to MRI: While MRI is widely considered the gold standard for diagnosing labral tears and various shoulder pathologies, ultrasound is a valuable complement. Its real-time and dynamic assessment capabilities are particularly beneficial when a patient's symptoms are best observed during specific movements or positions. This complementary role enhances the diagnostic accuracy and comprehensive evaluation of SLAP tears and related shoulder conditions [61].

Fluid Accumulation

Fluid accumulation within or around the shoulder joint, medically known as "shoulder effusion," is a common clinical concern that can result from various underlying causes. Ultrasound has emerged as a valuable diagnostic tool for identifying and characterizing these fluid collections, offering critical insights into a range of shoulder conditions [62]. One prevalent condition where ultrasound plays a crucial role is in diagnosing and monitoring bursitis. Bursitis involves the inflammation of bursae, which are fluid-filled sacs that reduce friction between tendons, ligaments, and bones. Ultrasound can effectively detect and visualize fluid buildup within the affected bursa, manifesting as an anechoic (dark) region on the ultrasound image. This characteristic finding of fluid within the bursa is instrumental in confirming the diagnosis of bursitis [63].

Similarly, ultrasound aids in assessing synovitis, a condition characterized by inflammation of the synovial lining of a joint. Synovitis often leads to increased synovial fluid within the joint space. Ultrasound imaging can reveal synovial thickening and excessive fluid, serving as diagnostic indicators of synovitis. Detecting synovitis is crucial as it can be associated with underlying conditions like rheumatoid arthritis or other inflammatory joint diseases [64]. Another significant application of ultrasound is identifying joint effusion fluid accumulation within the joint space. Joint effusion can arise from various causes, including injury, infection, or inflammatory conditions. Ultrasound can visualize this increased fluid within the joint, typically appearing as an anechoic or hypoechoic (dark or less bright) area within the joint cavity. Identifying joint effusion is vital for diagnosing and monitoring joint conditions [65].

In some cases, fluid accumulation within the shoulder joint may signify infection, such as septic arthritis. Ultrasound is pivotal in detecting fluid collections containing pus within the joint. This finding raises suspicion of an underlying infection and prompts further evaluation and treatment, emphasizing the diagnostic utility of ultrasound [66]. Furthermore, after fluid accumulation is identified, ultrasound-guided interventions are made possible beyond diagnosis. Healthcare providers can utilize ultrasound to locate and aspirate (remove) the accumulated fluid precisely or to guide the targeted administration of medications like corticosteroids to alleviate inflammation. This dual diagnostic and therapeutic capability underscores the importance of ultrasound in the comprehensive management of shoulder conditions associated with fluid accumulation [67].

Muscle and Soft Tissue Evaluation

Ultrasound imaging is versatile and indispensable when assessing various aspects of shoulder health. First, it excels in measuring muscle size and thickness around the shoulder joint. Muscle atrophy, the loss of muscle mass, can result from various causes, including disuse or nerve damage. By comparing the size of muscles on the affected side to those on the unaffected side, ultrasound can accurately detect muscle atrophy. This information is invaluable for diagnosing rotator cuff tears, nerve injuries, or muscle disorders [68]. Moreover, ultrasound is pivotal in identifying muscle tears or disruptions in muscle fibers. For instance, ultrasound can reveal discontinuities in the muscle tissue in rotator cuff tears involving muscles like the supraspinatus. Detecting muscle tears is vital for understanding the extent of the injury and guiding treatment decisions, which may encompass surgical intervention or conservative management [69].

Ultrasound extends its diagnostic prowess to evaluating tendons, including the rotator cuff tendons. It can detect various tendon pathologies such as tendonitis (inflammation of the tendons), tendinosis (degenerative changes in the tendons), and partial or complete tendon tears. Identifying these tendon abnormalities is pivotal for determining the most suitable course of treatment [70]. Additionally, ultrasound's sensitivity to soft tissue abnormalities is significant in shoulder assessment. It can detect cysts, masses, or foreign bodies around the shoulder joint, contributing to pain, restricted mobility, or other symptoms. Ultrasound identifies and characterizes these soft tissue anomalies, aiding in developing appropriate treatment plans [71].

Furthermore, ultrasound is a real-time guide for various medical procedures, such as injections or aspirations. For instance, when administering corticosteroid injections for conditions like shoulder impingement or bursitis, ultrasound ensures precision by helping healthcare providers accurately target the affected area during the procedure [72]. Lastly, ultrasound's dynamic evaluation capabilities are advantageous when assessing muscles and soft tissues during active movements. Patients can be instructed to perform specific shoulder movements, enabling healthcare providers to assess muscle function, detect movement-related abnormalities, and evaluate muscle contractions and coordination in real-time [73].

Guided Procedures

Ultrasound's real-time visualization capabilities offer a multitude of advantages in the realm of shoulder joint care and interventions. Its precision in targeting specific internal structures is a cornerstone of its utility. It enables healthcare providers to hone in on the area of interest within the shoulder joint and surrounding tissues. This level of accuracy is indispensable for both diagnostic and therapeutic procedures, ensuring that treatments are administered effectively and with precision [48]. For diagnostic aspirations, where the collection of fluid or tissue samples is necessary, ultrasound guidance proves invaluable. Whether evaluating suspected infections or inflammatory conditions in the shoulder joint, ultrasound allows healthcare providers to guide needles precisely, ensuring that samples are obtained from the desired location and aiding in accurate diagnoses [74].

In therapeutic interventions, ultrasound-guided injections of medications such as corticosteroids or local anesthetics are common practices for managing various shoulder conditions. This guidance system ensures that medications are delivered precisely to the affected area, maximizing their effectiveness while minimizing the risk of complications [75]. Moreover, ultrasound's real-time visualization contributes to patient comfort during procedures. Allowing providers to see needle placement as it happens reduces the likelihood of unintended tissue damage or discomfort associated with blind injections. Patients also benefit from immediate feedback, knowing that the procedure is being accurately performed, which can enhance their overall experience [76].

Following therapeutic injections, ultrasound plays a vital role in monitoring treatment response. Healthcare providers can assess changes in fluid accumulation, tissue inflammation, or other targeted conditions over time. This information is invaluable for making necessary adjustments to the treatment plan, ensuring that patients receive the most effective care [77]. Additionally, ultrasound-guided procedures enhance infection control by minimizing the risk of contamination or unintended needle placement, ultimately reducing the potential for infection associated with invasive procedures [78]. Lastly, involving patients in their healthcare journey is facilitated through ultrasound guidance. Patients can actively participate by viewing real-time images and understanding the treatment process more deeply. This engagement often improves patient compliance and satisfaction, fostering a collaborative approach to shoulder joint care [79].

Specific Findings in MRI

MRI is invaluable in diagnosing and assessing various shoulder conditions, providing detailed and precise information for healthcare professionals. It excels in characterizing rotator cuff tears, offering insights into their location, size, and thickness. This knowledge is pivotal for treatment planning, allowing for the differentiation between partial-thickness and full-thickness tears and guiding decisions on whether conservative management or surgical repair is the most appropriate course of action [80]. Furthermore, MRI proves highly effective in identifying labral tears, including specific types like SLAP tears, Bankart lesions, and posterior labral tears. It also aids in assessing shoulder instability, revealing indicators such as humeral head subluxations or dislocations, which are crucial for evaluating recurrent dislocation or instability episodes [81].

MRI's ability to visualize soft tissues and cartilage makes it an indispensable tool for evaluating arthritis in the shoulder. It can precisely depict cartilage loss, the presence of osteophytes (bone spurs), and the extent of synovial inflammation. Additionally, MRI assesses the integrity of articular surfaces and identifies joint space narrowing, facilitating the diagnosis and management of shoulder pain related to arthritis [82]. In addition to joint and tissue assessment, MRI can evaluate nerve and vascular structures within the shoulder region. It is instrumental in identifying nerve compression or vascular abnormalities contributing to shoulder symptoms, making it essential for diagnosing conditions like thoracic outlet syndrome or nerve impingement [43].

Moreover, MRI provides detailed insights into muscle and soft tissue pathologies around the shoulder, including muscle tears, atrophy, or edema. This information is invaluable for diagnosing and planning treatment for conditions such as muscle strains or inflammatory disorders affecting soft tissues [83]. In cases where shoulder pain may result from referred pain from other body areas or is linked to systemic conditions, MRI's utility extends beyond the shoulder joint. It aids in identifying underlying causes, such as pain originating from the cervical spine or conditions like rheumatoid arthritis [7]. For specific cases where enhanced visualization is required, MRI can utilize gadolinium-based contrast agents. This enhances the detection of vascular abnormalities, tumors, and inflammatory conditions, proving particularly useful in cases like vascular malformations, tumors, or inflammatory arthritis where contrast-enhanced imaging is essential for accurate assessment [84].

Treatment implications

Choice Between Surgical and Non-surgical Approaches

High-resolution imaging, such as MRI and ultrasound, helps healthcare providers determine whether a patient's shoulder condition requires surgical intervention or can be managed non-surgically. For instance, when diagnosing rotator cuff tears, imaging provides critical information about the tear's size, location, and extent. More minor, partial-thickness tears or less severe injuries may respond well to non-surgical treatments like physical therapy, anti-inflammatory medications, and corticosteroid injections. On the other hand, full-thickness tears or large tears often require surgical repair. Imaging is pivotal in this decision-making process, allowing healthcare providers to tailor the treatment plan to the specific condition [85].

Role of Imaging in Preoperative Planning

For patients requiring surgery, high-resolution imaging is instrumental in preoperative planning. Surgeons can use the imaging findings to determine the optimal surgical approach, plan the incision site, and understand the extent of tissue damage. In cases of labral tears or instability, for example, MRI can provide detailed insights into the type and location of the tear, enabling the surgeon to plan the appropriate repair technique. This precision enhances the surgical outcome and minimizes potential complications [86].

Monitoring Progression

High-resolution imaging also plays a role in monitoring the progression of shoulder conditions over time. It helps healthcare providers assess whether conservative treatments effectively manage the condition or if it is deteriorating. For instance, serial MRI scans can track changes in the rotator cuff tear size or the degree of cartilage loss in arthritis. This information guides treatment adjustments, ensuring patients receive the most appropriate care as their condition evolves [47].

Postoperative Assessment

After surgical intervention, imaging is essential for postoperative assessment. It allows healthcare providers to verify the procedure's success, evaluate the healing process, and detect potential complications. Postoperative imaging can confirm that structures like the rotator cuff or labrum have been adequately repaired and that there are no issues like excessive scarring or infection. This assessment determines when patients can begin rehabilitation and gradually return to normal activities [87].

Surgical versus non-surgical approaches

Non-surgical Approaches

Conservative management: In shoulder care, a conservative approach often takes precedence for various conditions, including mild to moderate rotator cuff tears, tendinitis, and bursitis. Initial recommendations may involve non-invasive methods such as rest, physical therapy, anti-inflammatory medications, and corticosteroid injections [88].

Image-guided injections: Precision is paramount when administering therapeutic injections, and high-resolution imaging, particularly ultrasound, plays a pivotal role in achieving this accuracy. By providing real-time guidance, ultrasound ensures that medications or treatments like platelet-rich plasma (PRP) are delivered to the precise location of concern within the shoulder joint, optimizing their therapeutic potential [89].

Progress monitoring: In the journey toward recovery, continuous assessment of the effectiveness of non-surgical treatments is essential. Both ultrasound and MRI serve as invaluable tools for monitoring progress over time. By providing detailed images and insights into the shoulder's condition, these imaging modalities aid clinicians in making informed decisions about treatment adjustments, ensuring that patients receive the most appropriate care tailored to their evolving needs [90].

Surgical Approaches

Rotator cuff repair: Surgical intervention is necessary in full-thickness rotator cuff tears, particularly when conservative treatments have proven ineffective. MRI is pivotal in assessing the tear's extent and precise location, providing surgeons with invaluable information for surgical planning and decision-making [91].

Labral repair: Complex conditions like SLAP tears may demand arthroscopic surgery for resolution. Here, MRI is a crucial diagnostic tool by offering comprehensive preoperative insights into the configuration and severity of the labral tear. This pre-surgical assessment is instrumental in guiding surgeons toward the most effective treatment strategies [92].

Joint replacement: In advanced stages of arthritis, considering joint replacement surgery, or arthroplasty, becomes a viable option. MRI proves indispensable in this context by allowing healthcare professionals to accurately evaluate the degree of joint degeneration. This information serves as a cornerstone in the planning and executing of joint replacement procedures, ensuring optimal patient outcomes [93].

Preoperative Planning With Imaging

MRI plays a pivotal role in shoulder surgery, offering a range of benefits for both preoperative planning and intraoperative guidance. One of its primary functions is surgical mapping, where MRI provides an intricate and comprehensive map of the shoulder joint. This map highlights critical details such as the location, size, and orientation of pathologies like rotator cuff tears, labral injuries, and arthritis. It offers surgeons invaluable insights to strategize their procedures effectively [94]. Beyond surgical mapping, MRI aids in tissue characterization, allowing surgeons to assess the condition of the surrounding soft tissues. This includes a detailed evaluation of muscles, tendons, ligaments, and cartilage, vital for planning precise surgical interventions and ensuring optimal postoperative outcomes [95].

Moreover, MRI contributes to risk assessment in shoulder surgery by uncovering any additional pathologies or anatomical variations that may impact surgical decisions. Such insights enable surgeons to tailor their approaches and strategies accordingly, ultimately enhancing the safety and success of the procedure [96]. MRI technology takes on an even more dynamic role in minimally invasive surgeries, such as arthroscopy. Real-time MRI or intraoperative MRI can assist surgeons in visualizing and navigating the surgical site with exceptional precision. This guidance ensures that the surgeon's actions are highly accurate, minimize risks, and maximize the procedure's effectiveness, ultimately benefiting patient outcomes [97,98].

Postoperative Assessment

Surgical outcomes: Following shoulder surgeries, the importance of postoperative imaging cannot be overstated. MRI plays a pivotal role in this regard as it allows clinicians to assess the procedure's success by revealing the status of repaired tendons, ligaments, or labra [99]. This postoperative evaluation is essential in determining whether the surgical intervention has effectively addressed the underlying issues.

Complication detection: High-resolution imaging techniques provided by MRI are instrumental in detecting and evaluating postoperative complications. These complications can range from infections to hematomas or hardware-related issues, and early detection is crucial for prompt intervention and improved patient outcomes [100].

Rehabilitation planning: Imaging is a valuable tool in guiding postoperative rehabilitation programs. By visualizing the post-surgical state of the shoulder joint, healthcare professionals can tailor rehabilitation plans to each patient's specific needs, ensuring optimal recovery and functional outcomes [101].

Advancements in high-resolution imaging

Emerging Technologies in Ultrasound

Ultrasound imaging continues to evolve with advancements that enhance its diagnostic capabilities and convenience. One notable development is adopting three-dimensional (3D) and four-dimensional (4D) ultrasound technology, which is becoming more accessible. This technology provides volumetric images of the shoulder joint, enabling a more comprehensive assessment of its complex structures and dynamic movements. It offers a valuable perspective for diagnosis and treatment planning [102]. Another significant innovation is shear wave elastography, a technique that measures tissue stiffness. This technology offers valuable insights into the elasticity of tendons, muscles, and other soft tissues within the shoulder joint. It proves particularly beneficial in diagnosing conditions like tendinitis and fibrosis, where tissue stiffness is crucial in assessment and management [103].

Contrast-enhanced ultrasound (CEUS) represents another noteworthy advancement. By utilizing contrast agents to enhance the visualization of blood flow within the shoulder joint, CEUS provides crucial information for assessing vascular abnormalities or tissue perfusion. This enhancement in imaging quality aids healthcare professionals in making more accurate assessments and decisions related to patient care [104]. Introducing portable and handheld ultrasound devices transforms point-of-care imaging across various clinical settings. These miniaturized and portable devices empower healthcare providers to conduct rapid assessments and interventions efficiently. Their versatility and accessibility are valuable for timely patient care, especially in emergencies [105]. Furthermore, artificial intelligence (AI) is increasingly significant in ultrasound imaging. AI algorithms are being developed to assist in image interpretation and diagnosis. These technologies promise to improve the accuracy and efficiency of ultrasound assessments, potentially reducing human error and enhancing the overall quality of patient care [106].

Advancements in MRI Techniques

The field of shoulder imaging has seen significant advancements in recent years, thanks to various MRI techniques that offer enhanced capabilities for diagnosis and assessment. High-field MRI scanners with stronger magnetic fields have emerged as a critical tool. These high-field systems have substantially improved image resolution and contrast, enabling healthcare professionals to delineate shoulder pathologies with unprecedented clarity [107]. Another invaluable technique is functional MRI (fMRI), encompassing methods like diffusion-weighted imaging (DWI) and diffusion tensor imaging (DTI). These techniques provide profound insights into tissue microstructure, allowing for the early detection of subtle changes in the shoulder joint that might indicate underlying issues [108].

Dynamic MRI, with its real-time imaging capabilities during shoulder movements, has become instrumental in assessing joint stability, identifying impingement problems, and evaluating labral function [109]. MR spectroscopy is yet another valuable approach, offering insights into the chemical composition of tissues within the shoulder joint and characterizing various pathologies [110]. The integration of multimodal imaging has proven indispensable for complex shoulder conditions, especially those involving suspected tumors or intricate issues. This approach combines MRI with other imaging modalities like positron emission tomography (PET) or computed tomography (CT) to provide a comprehensive assessment. By merging these imaging tools, healthcare providers can gain a more holistic understanding of shoulder conditions, facilitating better-informed treatment decisions [111].

Potential Future Developments

Quantitative imaging biomarkers: Ongoing research is dedicated to identifying quantitative imaging biomarkers, a critical endeavor that promises to revolutionize healthcare. These biomarkers hold the potential to predict disease progression and treatment response with remarkable precision, paving the way for highly personalized medicine approaches [112].

Integration with robotics: The convergence of high-resolution imaging technology with robotic-assisted surgical systems represents a cutting-edge advancement in medical procedures, especially for addressing shoulder joint conditions. This integration significantly enhances the precision and efficacy of minimally invasive interventions, promising improved patient outcomes [113].

Remote and telemedicine imaging: The rapid evolution of telemedicine technologies has ushered in a new era of healthcare accessibility. Innovations in remote image acquisition and interpretation are poised to transform healthcare delivery, particularly in underserved regions where specialized care is often scarce. This progress offers the prospect of bridging healthcare disparities and expanding access to medical expertise [114].

Advanced AI and machine learning: Artificial intelligence and machine learning algorithms continue to advance astonishingly. These technologies hold great promise for healthcare, particularly in medical imaging. They enable automated segmentation of anatomical structures, early detection of diseases, and significantly enhance diagnostic accuracy, ultimately improving patient outcomes [115].

Patient-centered imaging: The future of medical imaging is increasingly patient-centered. Tailoring imaging protocols to individual patient characteristics, such as age, gender, and clinical history, is poised to become the standard practice. This personalized approach promises to optimize the diagnostic process, ensuring that each patient receives the most appropriate and effective care [116].

Patient outcomes and prognosis

Impact of Imaging on Treatment Outcomes

Precision in treatment planning is paramount when addressing shoulder joint pathologies, and high-resolution imaging techniques like MRI and ultrasound play a pivotal role in achieving this. These imaging modalities offer clinicians a comprehensive view of the nature and extent of these pathologies, allowing for a more accurate diagnosis. This precision is invaluable as it empowers healthcare providers to tailor treatment plans to each patient's unique needs, ensuring that interventions are effective and well-suited to the condition at hand. Furthermore, the wealth of information obtained through imaging findings contributes significantly to informed decision-making. Patients and healthcare providers can make more educated choices when selecting surgical and non-surgical treatment options. This informed decision-making process increases the likelihood of successful outcomes and minimizes the risk of pursuing treatments that may not be suitable for a particular patient's condition.

When surgical interventions are necessary, imaging becomes even more critical. High-quality imaging assists in preoperative planning, enabling surgeons to meticulously assess the pathology, evaluate tissue quality, and determine the most appropriate surgical approach. This optimization enhances the precision of surgical procedures and, ultimately, leads to improved patient outcomes by minimizing the risk of complications and ensuring that the chosen surgical intervention is aligned with the patient's specific needs. Post-surgery imaging remains an invaluable tool for monitoring treatment efficacy. Regular follow-up imaging allows healthcare providers to track changes in the shoulder joint's appearance, helping them gauge whether the chosen treatment effectively addresses the underlying issue or if adjustments are required. This proactive approach ensures that patients receive timely modifications to their treatment plans, maximizing the chances of a successful recovery.

Finally, imaging also plays a crucial role in minimizing complications associated with shoulder joint pathologies and their treatments. By identifying potential complications early in the treatment process, healthcare providers can intervene promptly and implement strategies to mitigate risks, ultimately safeguarding the patient's well-being and optimizing their healthcare experience. In summary, high-resolution imaging is an indispensable tool in managing shoulder joint pathologies, offering precision in treatment planning, informed decision-making, optimized surgical interventions, ongoing treatment monitoring, and the reduction of potential complications.

Long-Term Prognosis and Follow-up

High-resolution imaging is indispensable in healthcare by enabling the comprehensive tracking of shoulder joint conditions as they evolve. It furnishes clinicians with a crucial baseline against which they can compare subsequent imaging studies, offering a window into the intricate course of the disease. Furthermore, this imaging technology is pivotal in the postoperative assessment of patients who have undergone surgical interventions, such as rotator cuff repairs or joint replacements. It serves as a reliable tool to affirm the success of these procedures, meticulously scrutinizing the integrity of repaired structures while vigilantly detecting any potential complications that may have arisen.

In rehabilitation, imaging charts the course for patients on their journey to recovery, guiding the formulation of tailored rehabilitation programs. Monitoring the healing process and evaluating the efficacy of prescribed exercises ensures that patients regain strength, range of motion, and functionality optimally. For chronic conditions like arthritis, high-resolution imaging is essential for long-term monitoring. Periodic assessments through imaging modalities allow healthcare providers to closely track the progression of joint degeneration, facilitating timely adjustments to treatment strategies as warranted. Furthermore, imaging findings serve as a valuable educational tool, empowering patients with insights into their current condition and potential future challenges, enabling them to make informed decisions about their care. Lastly, high-resolution imaging goes beyond mere observation; it also offers prognostic information, enlightening clinicians and patients about the likelihood of disease progression, the necessity for additional interventions, and the expected long-term outcomes. In this multifaceted role, high-resolution imaging truly emerges as an invaluable asset in healthcare, facilitating diagnosis, treatment, and patient empowerment.

Challenges and limitations

Challenges and limitations in high-resolution imaging adoption in healthcare revolve around cost, accessibility, interpretation variability, and concerns related to radiation exposure. Financial constraints, particularly associated with procedures like MRI scans, pose a significant hurdle, limiting access primarily to individuals with adequate insurance coverage or financial means. Geographic disparities further compound the issue, with rural and underserved areas experiencing restricted availability of advanced imaging technologies. In some healthcare settings, insufficient equipment can lead to extended wait times, reducing accessibility for patients needing prompt diagnosis and treatment.

Interpretation variability is a crucial consideration. For example, the quality of ultrasound images depends on the operator's skill, necessitating additional support for less-experienced operators. Even among seasoned radiologists or clinicians, interobserver variability can result in differing interpretations, impacting treatment decisions, especially in complex cases involving the shoulder joint. Radiation exposure is another concern, varying by modality. While MRI is generally considered safe due to the absence of ionizing radiation, issues with specific contrast agents, like gadolinium-based contrast agents, pose risks for individuals with impaired kidney function. Moreover, contrast-enhanced MRI may not be suitable for everyone due to allergies or contraindications, and the confined space of an MRI scanner can induce anxiety or claustrophobia, potentially requiring sedation with associated risks. Additionally, MRI may not be suitable for individuals with certain medical devices, limiting its use in specific populations. Addressing these barriers and concerns is imperative to ensuring equitable access and safe utilization of high-resolution imaging technologies in healthcare.

## Conclusions

In conclusion, high-resolution imaging, including ultrasound and MRI, is indispensable in diagnosing and managing shoulder joint pain. Through this comprehensive review, we have underscored its pivotal role in providing clinicians with detailed insights into the intricate structures of the shoulder, enabling accurate diagnosis and personalized treatment plans. The advantages of these imaging modalities, from real-time visualization to excellent soft tissue contrast, facilitate informed decision-making, optimize surgical procedures, and monitor patient progress. As we look to the future, advancements in technology, AI, and telemedicine promise to improve accessibility, accuracy, and patient outcomes in shoulder joint pain management. With its evolving capabilities, high-resolution imaging continues to empower healthcare providers to provide the highest quality care, ultimately enhancing the lives of those suffering from shoulder joint conditions.
